# General OOD detection via model-aware and subspace-aware variable priority

**DOI:** 10.1007/s10115-026-02872-5

**Published:** 2026-08-28

**Authors:** Min Lu, Hemant Ishwaran

**Affiliations:** Division of Biostatistics, Miller School of Medicine, University of Miami, Miami, USA

**Keywords:** Predictive subspace, Random forests, Survival analysis, Tabular supervised learning, Tree rules, Variable priority

## Abstract

Out-of-distribution (OOD) detection flags test inputs that depart from the data used to train a model. For structured tabular problems with regression or survival outcomes, existing methods remain limited because many OOD detectors are designed for classification or unstructured data. We introduce a tree based method that uses the rule structure of a supervised forest to determine the predictive subspace, the model-aware reference neighborhood, and the final OOD score in a single construction. The score compares each test input with forest selected reference cases only on prediction relevant variables, reducing signal dilution from nuisance coordinates. Across synthetic and real data benchmarks, the method performs especially well for subtle targeted feature shifts and changes in dependence. An esophageal cancer survival study further shows how OOD scores can reveal lymphadenectomy related shifts relevant to surgical guidelines.

## Introduction

Most prediction models are designed to return predictions, not to indicate when a new case is poorly supported by the data. A new input may differ from the training population in a single influential covariate, in a subtle combination of variables, or in the dependence structure among covariates, yet the model may still return a numerical prediction, survival curve, or risk score that may appear routine even though the test case should be considered suspicious. In settings such as clinical medicine and medical informatics, where predictions may influence diagnosis, monitoring, or treatment, this can have serious consequences. *Out-of-distribution* (OOD) detection addresses this problem by flagging inputs that are poorly represented by the data used to train the model [1, 2]. Because the forms of distributional departure that arise after deployment are rarely known in advance, an OOD detector must often be learned from *in-distribution* (ID) data alone.

We study OOD detection for supervised tabular prediction with continuous or time-to-event outcomes. Much of the OOD literature has been developed for image and text classification, where class probabilities, logits, and learned representations provide natural ingredients for OOD scoring. Regression and survival problems lack this class-based structure. When such problems are recorded in tabular form, however, the covariates are explicit, and the trained model can reveal how those covariates support prediction. The key idea in this paper is to use this model-derived structure to assess unusualness relative to the prediction task rather than relative to the covariate distribution alone. Specifically, we use the rule structure of a supervised random forest to identify coordinates that contribute to prediction and to isolate reference training cases that are locally relevant for a test input. The resulting OOD score compares the test input with those reference cases within the prediction-relevant covariate subspace.

To place this approach in the standard distribution-shift framework, write $\mathbb {P}_{X,Y}$ for the joint law of (*X*, *Y*) in the training population, with marginal law $\mathbb {P}_X$ and conditional law $\mathbb {P}_{Y\mid X}$. Let $\mathbb {P}^*_{X,Y}$ denote the corresponding test law, with marginal $\mathbb {P}^*_X$ and conditional law $\mathbb {P}^*_{Y\mid X}$. In the tabular settings considered here, $X=(X^{(1)},\ldots ,X^{(d)})\in \mathbb {R}^d$ is the structured covariate vector used to predict the outcome *Y*. The test distribution is OOD relative to the training distribution whenever $\mathbb {P}^*_{X,Y}\ne \mathbb {P}_{X,Y}$. Such a difference may arise through covariate shift, in which $\mathbb {P}_X$ changes while $\mathbb {P}_{Y\mid X}$ remains fixed; conditional or concept shift, in which $\mathbb {P}_{Y\mid X}$ changes; or changes in both components [3–8].

This taxonomy describes how training and test laws can differ, but it does not by itself determine which differences should make an individual input suspicious for a supervised predictor. A change in $\mathbb {P}_X$ along nonpredictive coordinates can make a case atypical in the full covariate space while leaving it well supported for prediction [9]. Conversely, a localized change involving a small number of influential variables, or a change in their dependence structure, may be difficult to recognize in the full covariate space but important for prediction. A supervised OOD score should therefore reflect how variables enter the prediction rule rather than treating every detectable departure in $\mathbb {P}_X$ as equally consequential [10]. Our score implements this principle by comparing each input with forest-selected training cases in the covariate subspace used for prediction.

Although this approach is very general, we note that a limitation of our method, shared by other OOD input methods, is that it cannot detect shifts that leave the covariate law unchanged. For a new case, our score uses only the case covariates and the fixed forest learned from the ID covariates and outcomes; no outcomes from the test distribution are observed when the score is computed. Consequently, a pure concept shift, with $\mathbb {P}^*_{Y\mid X}\ne \mathbb {P}_{Y\mid X}$ but $\mathbb {P}^*_X=\mathbb {P}_X$, is OOD under the joint law but leaves no observable covariate signal for the detector. Detecting such a shift requires outcomes from the test distribution or additional assumptions, and is outside the scope of this paper.

### Predictive subspace

We formalize the part of the covariate space relevant to prediction using a predictive subspace. Let $S_0 \subset \{1,\ldots ,d\}$ denote a set of coordinates through which the training conditional law factors. For any $x\in \mathbb {R}^d$, write $x^{(S_0)}=(x^{(j)})_{j\in S_0}$. We suppose

$$ \mathbb {P}(Y\in A\mid X=x) = \mathbb {P}(Y\in A\mid X^{(S_0)}=x^{(S_0)}) $$

for every measurable set *A* and $\mathbb {P}_X$-almost every *x*. Thus, under the training distribution, the conditional law of *Y* depends on *X* only through $X^{(S_0)}$. We do not require $S_0$ to be unique or minimal. Any set satisfying this condition provides a valid population target, although the purpose of the restriction is to remove coordinates that are unnecessary for prediction.

The set $S_0$ describes the population target of the subspace restriction, but it is unknown in practice. Our method uses variable priority from a supervised forest to construct an empirical signal set $\widehat{S}$ and then uses the same forest to determine the local reference set for a test input. Thus, $S_0$ motivates the subspace restriction, whereas $\widehat{S}$ is the learned subspace used by the score.

### Approach

The proposed method rests on two properties. First, the OOD score depends on the trained supervised model, not only on the geometry or density of the covariates. Second, once the relevant training cases are identified, the final comparison uses only coordinates containing predictive information.

The terms *model-aware* and *subspace-aware* refer to these two operational properties. Fix a training procedure $\mathcal {P}$ and hold any internal algorithmic randomness fixed. Given training data $\mathcal {L} = \{(x_i, y_i)\}_{i=1}^n$, write $\hat{f} = \mathcal {P}(\mathcal {L})$ and $x_{1:n} = (x_1, \ldots , x_n)$.

#### Definition 1

*(Model-aware OOD score)* An OOD score $d(x^*; x_{1:n}, \hat{f})$ is *model-aware* if the trained supervised model $\hat{f}$ enters the construction of the score. Thus, holding $x^*$ and $x_{1:n}$ fixed, changing the training outcomes can change the score through $\hat{f}$.

#### Definition 2

*(Subspace-aware OOD score)* Let $S \subset \{1,\ldots ,d\}$ be a selected set of coordinates, and let $\mathcal {R}(x^*; x_{1:n}, \hat{f}) \subseteq \{x_1,\ldots ,x_n\}$ denote the reference set used for test input $x^*$. An OOD score is *subspace-aware with respect to S* if, after the reference set has been formed, the final discrepancy depends on $x^*$ and the reference points only through their coordinates in *S*.

Our construction satisfies both properties with a single learned object. We train a supervised forest on the ID data and use its decision rules in two connected ways. First, the rules identify the empirical signal set $\widehat{S}$. Second, for a test point $x^*$, we relax the rules containing $x^*$ one selected coordinate at a time, rank the training points, and form a neighborhood $\mathcal {N}_K(x^*)$. This neighborhood is the reference set $\mathcal {R}(x^*; x_{1:n}, \hat{f})$ in Definition 2. The final OOD score averages the distance from $x^*$ to this reference set, computed only on $\widehat{S}$,

$$ d(x^*) = \frac{1}{K} \sum _{z \in \mathcal {N}_K(x^*)} D_{\widehat{S}}(x^*, z), $$

where $D_{\widehat{S}}$ denotes a distance using only the coordinates in $\widehat{S}$, with optional weights reflecting their relative importance. Thus, the score depends on $\hat{f}$ through both $\widehat{S}$ and $\mathcal {N}_K(x^*)$, satisfying Definition 1, while its final discrepancy uses only $\widehat{S}$, satisfying Definition 2.

### Main contribution and evaluation

Our main contribution is an integrated model-aware and subspace-aware OOD score for tabular supervised learning. A single trained supervised forest identifies prediction-relevant variables, constructs a local reference set for each test input through learned rule regions, and computes a weighted distance on the selected variables. This construction differs from approaches that first reduce dimension or select features and then apply a standard distance, nearest-neighbor, or density-based anomaly score.

The empirical sections evaluate the construction in three ways. First, matched ablations separate the effects of variable priority selection, the forest rule neighborhood, and variable weighting. Second, benchmark experiments compare the method with existing OOD detectors across synthetic, regression, and high-dimensional survival settings. Third, an esophageal cancer case study shows how the scores inform a clinical survival analysis. Software implementing the method is available in the CRAN package varPro. Public benchmark datasets and microarray data used in the experiments are available from the cited references.

### Organization

The paper is organized as follows. Section 2 reviews related work and explains how our approach differs. Section 3 develops the methodology, and Section 4 describes the comparison methods. Section 5 presents the benchmarking results. Section 6 applies the method to a survival analysis of esophageal cancer. Section 7 summarizes and discusses limitations.

## Related work

Research on out-of-distribution (OOD) behavior spans anomaly detection, open-set recognition, selective prediction, domain generalization, and OOD detection [2, 11–13]. Recent surveys distinguish methods developed for supervised learning [2, 11, 12] from methods tailored to structured domains such as graphs and time series [14–16]. Our focus is test time OOD scoring for supervised tabular models, especially regression and survival analysis, where the score should use what the fitted model has learned about prediction relevant covariates.

### Dataset shift and prediction relevance

Dataset shift is commonly described as a difference between the joint training and test distributions. Standard taxonomies distinguish changes in the covariate distribution from changes in the conditional law of the outcome [3, 4, 7]. Covariate shift refers to a change in $\mathbb {P}_X$ with $\mathbb {P}_{Y\mid X}$ stable, while concept shift refers to a change in the conditional relationship between predictors and outcome [5–7]. Domain adaptation theory gives related bounds for learning when training and test distributions differ [8].

In supervised tabular prediction, a covariate departure is most relevant when it occurs in directions that affect the fitted prediction rule. Departures confined to nuisance coordinates may make a point unusual under $\mathbb {P}_X$ without making it unusual for the prediction task. This motivates an OOD score that uses the fitted model to determine both the variables and the local training cases used in the comparison.

### Model based OOD scores

Most OOD detection methods were developed for classification, where class probabilities, logits, and learned representations provide natural scoring rules. Examples include maximum softmax probability [1], ODIN [17], energy-based scores [18], Mahalanobis scores in learned feature space [19], deep nearest neighbors [20], and posterior or representation shaping methods [21–23]. Related subspace and spectral methods decompose or project learned activations before scoring [24–26]. These approaches can be highly effective for image and text benchmarks, but they are closely tied to discrete labels, deep representations, or global feature spaces, and are not directly designed for continuous outcomes or survival data.

For regression and related continuous outcome problems, OOD detection has often followed an uncertainty based paradigm. Deep ensembles [27], Monte Carlo dropout [28], evidential regression [29], prior networks [30], predictor entropy methods [31], Gaussian processes, and deep kernel learning [32–34] use predictive uncertainty as an OOD signal. These methods are model-aware, but the resulting scores are usually global in the feature space and do not explicitly separate departures in predictive variables from departures in nuisance variables.

### Tabular, geometric, and calibrated detectors

A separate line of work screens for unusual test inputs using the covariate distribution alone. Standard tabular baselines include distance based outlier scores and *k*-nearest-neighbor criteria [35, 36], local outlier factor [37], one-class support vector machines [38], and tree partition scores such as Isolation Forest [39]. These methods are useful general anomaly detectors, but they do not use the supervised response and therefore do not distinguish departures in variables that matter for prediction from departures in variables that do not.

Recent tabular and clinical studies highlight the practical importance of this issue. ODROP trains a separate OOD reject option for disease onset prediction [40], while the Chameleons benchmark studies OOD behavior in medical tabular data [41]. Broader medical OOD reviews also emphasize the need for accurate and interpretable detection in clinical settings [42, 43]. These studies show the importance of OOD detection for tabular risk prediction, but they do not provide a model-aware, subspace-aware score for regression or survival models.

Conformal methods offer a complementary perspective. Given a nonconformity score, conformal prediction can provide calibrated *p*-values, intervals, or testing procedures [44–48]. Such methods can calibrate OOD decisions once a score has been chosen. Our focus is different. We design the score itself, and leave conformal calibration of the proposed score for future work.

### Positioning

Most existing methods emphasize one of two properties. Some are model-aware because the score depends on a fitted predictor trained on labeled data. Others are subspace-aware because the score is computed after restricting attention to selected variables or learned feature directions. In the tabular regression and survival settings considered here, these properties are rarely combined. Classification and deep representation methods are often model-aware but are not designed for continuous outcomes or survival analysis. Classical tabular anomaly detectors work directly with *X* and are not model-aware. Uncertainty based regression methods use the fitted model, but usually score globally in the full feature space.

The proposed method is designed for this gap. It is model-aware because the score is built from a supervised forest trained on labeled data, and subspace-aware because the final comparison is restricted to variables selected as task relevant. The next section gives the construction.

## Methodology

We now give the construction of the proposed method. Given labeled training data $(x_1,y_1),\ldots ,(x_n,y_n)$, we first fit a random forest [49]. For a test input *x*, the forest is then used to construct two objects needed for scoring. The first is a signal set $S \subset \{1,\ldots ,d\}$ defining the coordinates on which *x* is compared with the training data. The second is a local reference neighborhood $\mathcal {N}_K(x)$ defining the training cases to which *x* is compared. The final score averages a distance from *x* to this neighborhood using only the coordinates in *S*.

Random forests are a natural choice for our approach because they provide a common framework for regression, classification, and survival outcomes, and because their learned partitions group observations with similar outcome behavior. This partition based notion of similarity is commonly summarized by random forest proximity, where two inputs are considered similar if they often fall in the same terminal nodes across trees. Our construction however uses the forest differently. Rather than using terminal node co-occurrence itself as the OOD score, we use the learned rule structure of the forest to build the signal set and the local reference neighborhood.

Each tree can be written as a collection of decision rules. A rule is a path from the root to a terminal node, expressed as simple conditions on individual variables, for example $x^{(1)} > 3$ and $x^{(5)} < 1$. These conditions define a rectangular region of the input space. Across the ensemble, the full collection of rules describes how regions of the covariate space are associated with outcome patterns. The rules are used in two connected steps. First, they are analyzed to identify variables carrying predictive information, yielding a signal set $S \subset \{1,\ldots ,d\}$. Second, for a test input *x*, the rules that contain *x* are relaxed one selected variable at a time to identify training points that repeatedly co-occur with *x* under the forest. These co-occurrences rank the training points and form the reference neighborhood $\mathcal {N}_K(x)$. The final OOD score is the average distance from *x* to this neighborhood, restricted to the signal coordinates, $d(x) = \sum _{z \in \mathcal {N}_K(x)} D_S(x,z)/K$.

The remainder of this section develops each component in turn. A standalone summary is given in Algorithm 1, which we call *OutPro* (OOD using variable priority). For a test input $x=(x^{(1)},\ldots ,x^{(d)})\in \mathbb {R}^d$, OutPro takes the labeled training data and trained forest as input and returns a scalar OOD score *d*(*x*), with larger values indicating greater departure from the training distribution in the predictive subspace.

### Variable priority and selection of *S*

Both neighborhood construction and distance evaluation depend on a set of task relevant variables

$$ S = \{s_1,\ldots ,s_q\}\subset \{1,\ldots ,d\}. $$

We estimate *S*, together with weights $\{w_s\}_{s \in S}$ measuring the relative strength of each selected coordinate, using Variable Priority (VarPro) [50–53]. VarPro is a model based feature selection method derived from the rule regions produced by a forest. For a rule region, the release region for variable *s* is the expanded region obtained by removing the constraint on *s* while keeping all other bounds fixed.

A variable receives high priority when releasing its constraint consistently changes the outcome behavior of the training points in the region. Variables whose release has little effect are treated as noise. Thus VarPro scores variables by outcome changes under rule relaxation, rather than by split frequency or tree usage alone. This helps distinguish genuine predictors from correlated surrogates and is directly relevant for OOD scoring. Including noise variables in *S* dilutes the distance calculation across uninformative dimensions, whereas excluding signal variables can hide departures in the predictive subspace.

**Fig. 1:**
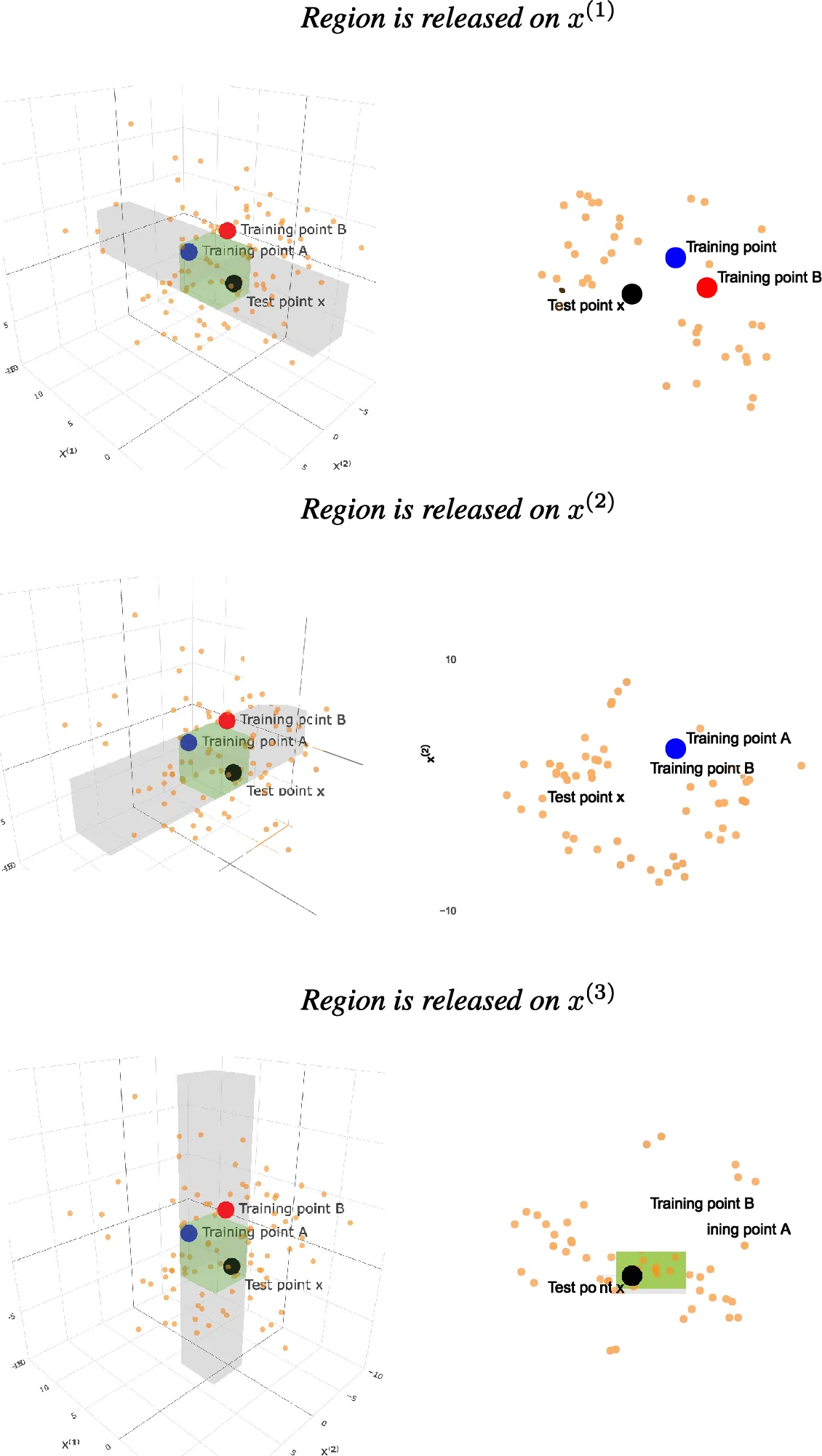
Release-region mechanism used for frequency profiling. The green cube is a rule containing the test point *x* shown in black, and grey cuboids are release regions formed by removing one coordinate constraint at a time. Training points A and B are equidistant from *x* in ordinary Euclidean distance, but their release profiles differ. A appears in all three release regions, giving counts (1, 1, 1), whereas B appears only after releasing $x^{(2)}$ and $x^{(3)}$, giving counts (0, 1, 1). Thus A is more similar to *x* under the rule relaxation profile

### Frequency profiling via rule relaxation

The same release operation is used to build the reference neighborhood for a test input *x*. Let $\mathcal {R}(x)$ denote the set of decision rules from the forest whose terminal regions contain *x*. Each rule $\zeta \in \mathcal {R}(x)$ defines a hyperrectangle $R(\zeta ) \subset \mathbb {R}^d$. For each selected coordinate s∈S, let $R(\zeta ^s)$ be the release region formed by removing the constraint on *s* and retaining all other bounds.

**Algorithm 1:**
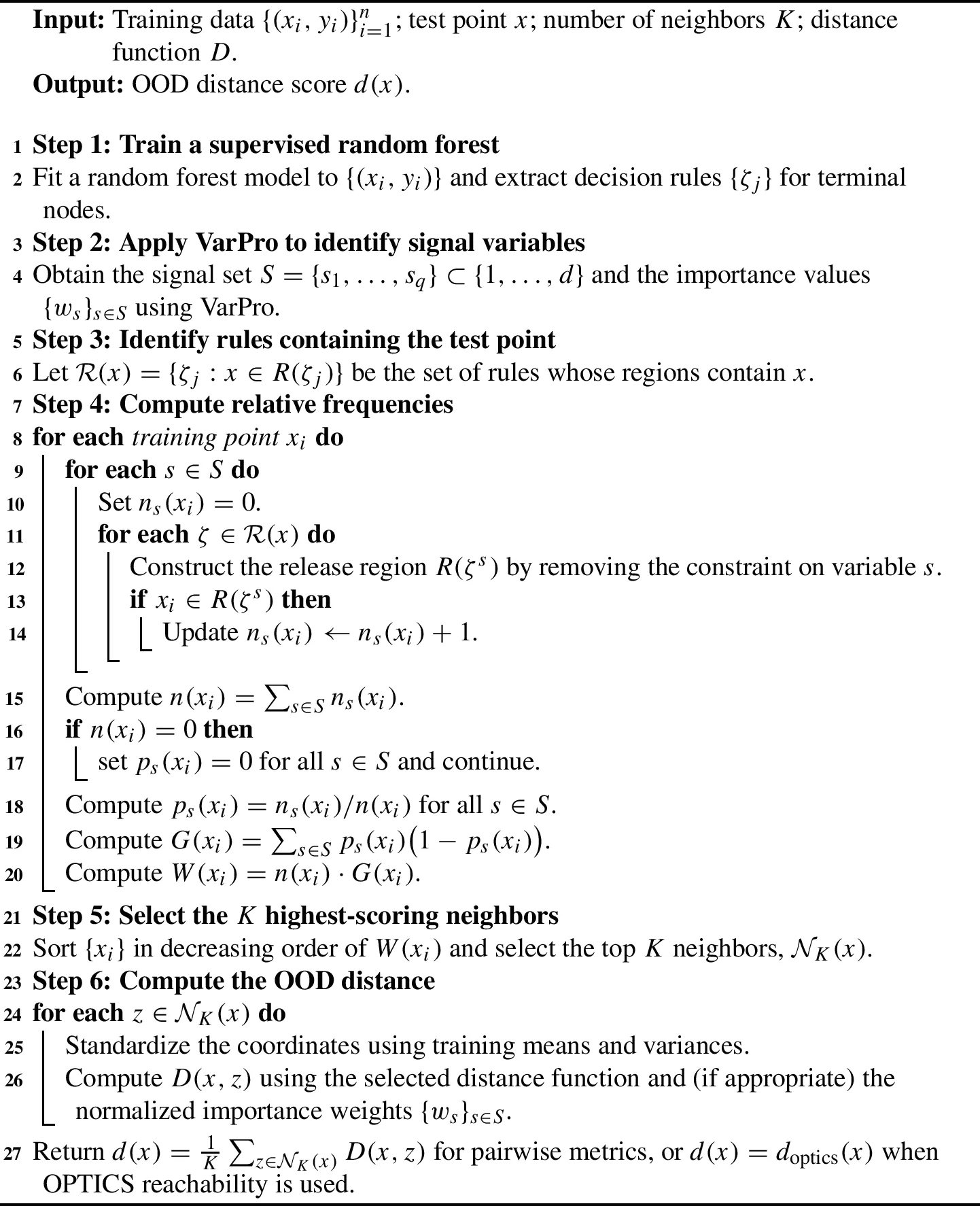
OOD using variable priority (OutPro)

For each training point $x_i$, we count its appearances in these release regions. For s∈S, define

$$ n_s(x_i) = \sum _{\zeta \in \mathcal {R}(x)} I\{x_i \in R(\zeta ^s)\}, $$

and let

$$ n(x_i) = \sum _{s \in S} n_s(x_i). $$

When $n(x_i)>0$, the frequency profile of $x_i$ relative to *x* is

$$ (p_{s_1}(x_i), \ldots , p_{s_q}(x_i)) = \left( \frac{n_{s_1}(x_i)}{n(x_i)}, \ldots , \frac{n_{s_q}(x_i)}{n(x_i)} \right) . $$

If $n(x_i)=0$, we set $p_s(x_i)=0$ for all s∈S and exclude $x_i$ from neighborhood construction. The profile records how often a training point co-occurs with *x* after releasing each selected coordinate, and therefore summarizes its alignment with *x* under the forest’s local rule structure.

Figure 1 illustrates this construction for a single rule and three selected coordinates. Points A and B are equidistant from *x* in ordinary Euclidean distance, but their release profiles differ. Point A appears in all three release regions, whereas point B appears in only two. The frequency profile therefore captures local similarity under the forest’s rule structure, rather than under a fixed Euclidean metric.

### Proximity scoring from frequency dispersion

This subsection defines a proximity score $W(x_i)$ for each training point $x_i$, relative to the test input *x*. The score is used to rank training points before the final OOD distance is computed. A training point receives a large value of $W(x_i)$ when it appears often in the release regions for *x* and when those appearances are spread across the selected variables in *S*. A point receives a smaller value when it appears rarely, or when its appearances are concentrated in release regions for only a few selected variables.

For a training point $x_i$ with $n(x_i)>0$, recall that $p_s(x_i)$ is the relative frequency with which $x_i$ appears after releasing coordinate s∈S. We summarize the balance of this profile using the Gini impurity

$$ G(x_i) = \sum _{s \in S} p_s(x_i) \big (1 - p_s(x_i)\big ). $$

Larger values occur when co-occurrence is spread more evenly across the selected coordinates. The final proximity score also accounts for the total number of appearances,

$$ W(x_i) = n(x_i) G(x_i). $$

Thus $W(x_i)$ favors training points that both appear frequently in release regions and have balanced co-occurrence across the predictive coordinates.

### OOD distance via subspace distance functions

The reference neighborhood for *x* is the set $\mathcal {N}_K(x)$ of the *K* training points with the largest proximity scores $W(x_i)$. The OOD score is then the average distance from *x* to this neighborhood,

$$ d(x) = \frac{1}{K} \sum _{z \in \mathcal {N}_K(x)} D(x,z). $$

The distance *D*(*x*, *z*) is computed only on the selected variables s∈S and is weighted by the VarPro scores $\{w_s\}_{s \in S}$. The weights are positive and normalized, so higher priority variables receive greater emphasis and lower priority variables are down-weighted. Several different types of distance functions are used in the empirical experiments; these are conveniently summarized in Table 1.

**Table 1:** Distance functions used in the experiments. Coordinates are standardized using training means and variances, and all distances are restricted to the selected coordinates *S*. Except for Mahalanobis distance, each coordinate s∈S is weighted by its normalized VarPro weight $w_s$. The first five rows define pairwise distances *D*(*x*, *z*) used in the averaged OOD score, while OPTICS defines a direct reachability score for *x*

Distance	Definition
Product	$\displaystyle D_{\text {prod}}(x,z) = \prod _{s \in S} \left( |x^{(s)}-z^{(s)}|+\varepsilon \right) ^{w_s}$, with ε>0 used for numerical stability.
Euclidean	$\displaystyle D_{\text {euclid}}(x,z) = \left\{ \sum _{s \in S} w_s (x^{(s)}-z^{(s)})^2 \right\} ^{1/2}.$
Manhattan	$\displaystyle D_{\text {manh}}(x,z) = \sum _{s \in S} w_s |x^{(s)}-z^{(s)}|.$
Minkowski	$\displaystyle D_{\text {mink}}(x,z) = \left\{ \sum _{s \in S} w_s |x^{(s)}-z^{(s)}|^p \right\} ^{1/p}.$ We use p=4 in the empirical studies.
Mahalanobis	Let $\Sigma ^{(S)}$ be the empirical covariance matrix of the training data restricted to *S*. Then $\displaystyle D_{\text {mahal}}(x,z) = \left\{ (x^{(S)}-z^{(S)})^T (\Sigma ^{(S)})^{-1} (x^{(S)}-z^{(S)}) \right\} ^{1/2}.$
OPTICS	OPTICS [54] is applied to $\mathcal {N}_K(x)\cup \{x\}$ after restricting to *S* and scaling coordinate *s* by $\sqrt{w_s}$. The OOD score is the reachability value $\displaystyle d_{\text {optics}}(x) = \text {Reachability} \big (x \mid \mathcal {N}_K(x), \texttt {minPts}, \{w_s\}_{s \in S}\big ).$ We use the optics function from the R package dbscan with minPts=5 [55].

## Comparison methods

We compare OutPro with OOD detectors that are applicable to continuous outcomes and have reliable open source implementations. The baselines include uncertainty based methods, latent representation methods, gradient based scores, and classical unsupervised tabular detectors. The unsupervised methods use only the training covariates, whereas the model based methods use a fitted predictor. Table 2 summarizes the score and implementation used by each comparison method.

All neural network methods share the same base architecture, implemented with the Keras API [56]. The network has two hidden ReLU layers, batch normalization, dropout, and a single output node trained by mean squared error. MSP, ODIN, Energy, Sensitivity, DKL, GMM, and latent space *k*-nearest neighbors are computed from this shared fitted network, as described in Table 2. Conformal prediction and deep ensembles use the same architecture but refit it in their own split calibration and ensemble settings. Gaussian process methods use the laGP package [32, 57]. All scores are oriented so that larger values indicate stronger evidence of OOD behavior.

**Table 2:** Comparison methods. Here *f*(*x*) is the fitted prediction, *h*(*x*) is the penultimate layer representation of the neural network, $\bar{y}_{\textrm{train}}$ is the mean training response, and $\tilde{x}=x+\epsilon \operatorname {sign}(x)$ with ϵ=0.01. Input space methods use standardized covariates unless noted otherwise.

Method	OOD score or implementation summary
MSP [1]	Regression adaptation using prediction deviation, $|f(x)-\bar{y}_{\textrm{train}}|$. This is monotone equivalent to the reciprocal confidence score used in the implementation.
ODIN [17]	Regression adaptation using the perturbed input $\tilde{x}$ and score $|f(\tilde{x})-\bar{y}_{\textrm{train}}|$.
Energy [18]	Perturbation based latent score $\Vert h(\tilde{x})\Vert _2^2$.
Mahalanobis	Input space Mahalanobis distance $\{(x-\mu )^T\Sigma ^{-1}(x-\mu )\}^{1/2}$, where μ and Σ are estimated from the training covariates.
LOF [37]	Local outlier factor computed on standardized covariates with k=min(20,n-1).
Isolation Forest [39]	Isolation Forest anomaly score using 200 trees and subsample size min(256,n).
OCSVM [38]	One class support vector machine with radial kernel $K(x,x')=\exp \{-\gamma \Vert x-x'\Vert _2^2\}$, γ=1/d. The OOD score is the negative fitted decision function. The parameter ν is set to the benchmark cutoff rate, truncated to [1/max(2,n),0.5].
kNN density [35, 36]	Robust *k*-nearest-neighbor density score. Covariates are centered by the training median and scaled by the training median absolute deviation. The score is the log median radius to the k=min(20,n-1) nearest training points.
Conformal prediction [44]	Split conformal score using 75% of the training data for fitting and 25% for calibration. The ranking score is $|f(x)-\operatorname {median}(f_{\textrm{calib}})|$; the binary decision compares this value with the corresponding calibration quantile.
Gaussian process	Local approximate Gaussian process regression using laGP; the OOD score is the posterior predictive variance at *x*.
Sensitivity [58]	Average absolute input gradient, $d^{-1}\sum _{j=1}^d |\partial f(x)/\partial x^{(j)}|$.
DKL [33]	Deep kernel learning baseline. A Gaussian process is fit by laGP to the neural representation *h*(*x*), and the OOD score is the posterior predictive variance.
Deep ensembles [27]	Variance of predictions across independently initialized neural network ensemble members.
GMM [59]	Gaussian mixture density in PCA reduced latent space. The OOD score is $-\log p(\tilde{h}(x))$, where $\tilde{h}(x)$ is the reduced representation.
DkNN [20]	Latent space *k*-nearest-neighbor score, $k^{-1}\sum _{j=1}^k\Vert h(x)-h(x_{(j)})\Vert _2$, using nearest neighbors in the neural representation.

## Benchmarking OOD detection methods in regression

We evaluate performance using a combination of simulated and real regression problems. On synthetic data, we use a well-known machine learning model with independent covariates (Sect. 5.1), which provides a controlled anomaly framework with clearly defined ground-truth labels. For real data, we consider two sets of experiments. The first is a large collection of regression benchmarks (Sect. 5.3) with varying sample sizes, dimensions, and signal-to-noise ratios. The second uses high-dimensional microarray survival studies (Sect. 5.4), where the number of features greatly exceeds the sample size. For the real-data experiments, anomalies are generated using a copula-based strategy described in Sect. 5.2, which allows targeted perturbations of marginal distributions and joint dependence. Test cases are drawn from a held-out subset of the data, and anomalous inputs are created by perturbing selected features to simulate OOD behavior.

### Friedman simulation

We first consider a simulated regression setting based on the well-known Friedman model [60]. Each covariate $X^{(j)} \sim U[0,1]$ independently for j=1,…,d. The response is generated according to

$$ Y = 10 \sin \big (\pi X^{(1)} X^{(2)}\big ) + 20 \big (X^{(3)} - 0.5\big )^2 + 10 X^{(4)} + 5 X^{(5)} + \varepsilon , $$

where $\varepsilon \sim N(0, \sigma ^2)$ and the remaining d-5 covariates are irrelevant noise features. We use d=20 and σ=1.

To simulate OOD instances, we apply a fixed additive shift to a subset of features identified as predictive of the outcome. Although the true signal variables are known in this simulation, we use tree-based gradient boosting [61] to rank features by their contribution to predictive accuracy. This data driven approach to feature selection mirrors the methodology used in our later real-data experiments and ensures consistency across settings. From the ranked list, the top ten percent of features are selected, and each is perturbed by a fixed additive amount scaled by its standard deviation, with direction randomly assigned for each test point.

#### Ground truth

The additive shift procedure is a common strategy in OOD detection research. However, the labeling rule that defines a shifted point as anomalous has a subtle issue that is often overlooked: a shifted point may lie entirely within the support of the training distribution and therefore represent a perfectly valid input.

To see this, note that under the Friedman setup the covariates $X^{(j)}$ are independent and uniformly distributed on [0, 1]. Applying an additive shift $\delta = (\delta _1, \ldots , \delta _d)$ to the covariate vector $X = (X^{(1)}, \ldots , X^{(d)})$ produces a perturbed point

$$ X^* = X + \delta . $$

Because the covariates are independent with bounded support, a shifted point $X^*$ becomes anomalous only if at least one coordinate falls outside the unit interval. For coordinate *j*, the condition $X^{(j)} + \delta _j \in [0,1]$ is equivalent to

$$ -\delta _j \le X^{(j)} \le 1 - \delta _j. $$

Hence the probability that all coordinates remain within the original hypercube is

$$ \mathbb {P}(\text {all coordinates inside}) = \prod _{j=1}^d (1 - |\delta _j|)_+, $$

where $(a)_+:= \max (a, 0)$. The true fraction of shifted points that fall outside the support is therefore 1 minus this value, which for small shifts can be approximated to first order as $\sum _{j=1}^d |\delta _j|$ and can therefore be substantially less than 1.

Therefore many nominally shifted points may still lie entirely within the original support and are therefore legitimate data values, particularly when shifts are small or the dimension is moderate. To avoid this ambiguity, we adopt the rule that a point is labeled anomalous if and only if at least one of its coordinates falls outside [0, 1].

#### Experimental settings

We generated 100 independent datasets of size n=2000 from the Friedman model. Each dataset was randomly split into 80% training and 20% testing and anomalies were created by perturbing test points using shifts as described above. Shifts were applied to the top predictive features ranked by gradient boosting and scaled by the empirical standard deviation of each feature. We considered five shift magnitudes: 0.05, 0.25, 0.5, 1.0, and 2.0. Ground-truth labels were defined by classifying a perturbed test point as anomalous if and only if at least one coordinate fell outside [0, 1].

#### Results

**Fig. 2:**
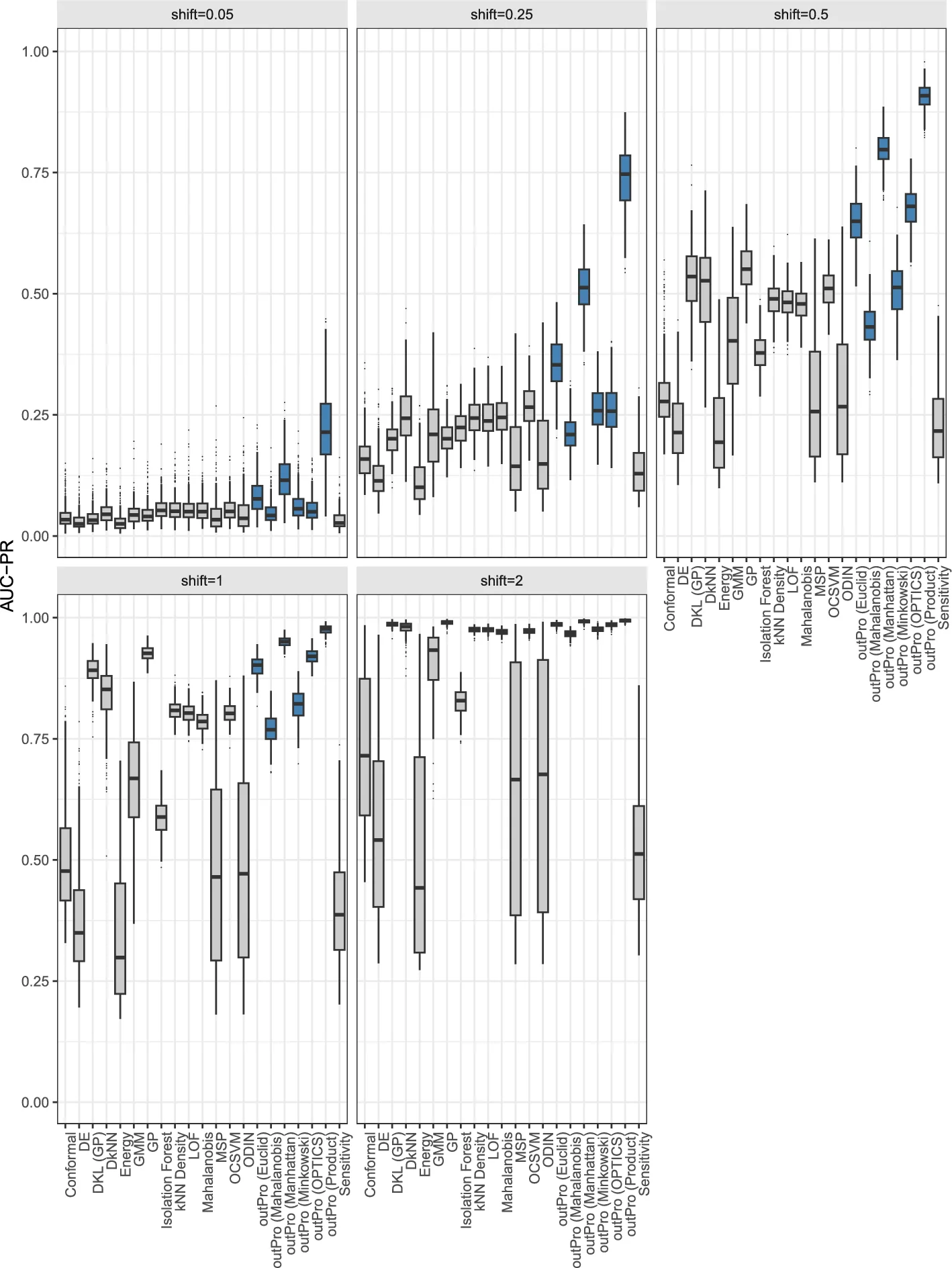
Boxplots of AUC-PR values for each method across 100 runs at varying shift magnitudes in the Friedman simulation. Blue indicates OutPro methods. Performance improves for all methods as the shift magnitude increases. The OutPro product and Manhattan scores are best for small shifts (0.05 to 0.5), where detection is most difficult

**Fig. 3:**
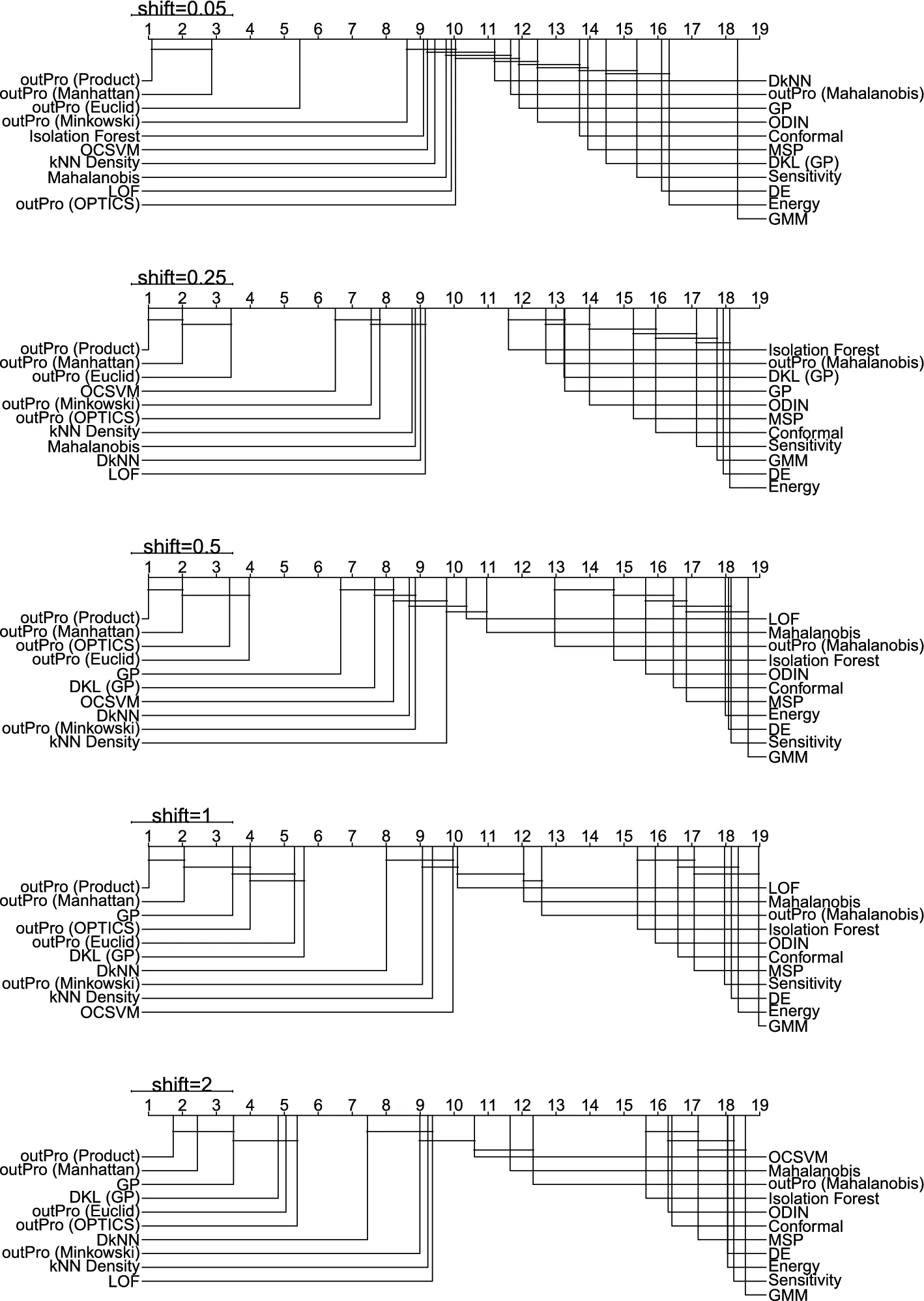
Critical difference (CD) plots comparing average AUC-PR rankings for the Friedman simulation at each shift magnitude. Lower ranks indicate better performance. Methods with no statistically significant difference at α=0.05 are connected by a horizontal bar. For shifts 0.05, 0.25, and 0.5, the OutPro product and Manhattan scores rank near the top, while the best classical tabular baselines are in the next tier. The product score has the best average rank at every shift magnitude

Performance was measured by the area under the precision-recall curve (AUC-PR). Boxplots in Figure 2 show that the OutPro procedures are among the best for small shifts (0.05 to 0.5), which is the setting where anomalies are subtle and remain close to the training distribution. To systematically compare methods, critical difference (CD) plots are given in Figure 3, with significance assessed using the Nemenyi test. The OutPro product score attains the best average rank at every shift magnitude, and Manhattan is consistently second. For the smallest shifts, the best baselines are the classical tabular methods, especially Isolation Forest, OCSVM, robust *k*NN density, LOF, and the global Mahalanobis score, but they do not overtake OutPro. As the shift grows, GP and DKL (GP) improve and, together with DkNN, become competitive with OutPro. Isolation Forest is less effective for the larger shifts, while robust *k*NN density and LOF remain in the middle. Overall, the results show that density based tabular baselines can be competitive when the shift is large enough to create obvious low density points, but OutPro is best when the perturbation is small and functionally targeted.

### Copula-based strategy for anomaly generation

The Friedman simulation provides a controlled benchmark with independent features and bounded support. Real tabular data are more complex, with skewed or heavy-tailed marginals and nontrivial dependence among variables. To generate realistic anomalies in this setting, we use a copula-based strategy that separates marginal behavior from dependence and perturbs each component in a controlled way.

Let $X=(X^{(1)},\ldots ,X^{(d)})$ be a continuous random vector with marginal CDFs $F_1,\ldots ,F_d$. Applying the probability integral transform coordinatewise gives

$$ U^{(j)} = F_j(X^{(j)}), \quad j=1,\ldots ,d. $$

For continuous marginals, each $U^{(j)}$ is uniform on [0, 1], and the joint distribution of $U=(U^{(1)},\ldots ,U^{(d)})$ is the copula *C*. By Sklar’s theorem [62],

$$ F(x^{(1)},\ldots ,x^{(d)}) = C\{F_1(x^{(1)}),\ldots ,F_d(x^{(d)})\}. $$

Thus a point on the copula scale can be mapped back to the data scale by $x^{(j)}=F_j^{-1}(u^{(j)})$. The marginal inverse CDFs determine the coordinate scales, while the joint arrangement of the *u* values determines dependence.

**Fig. 4:**
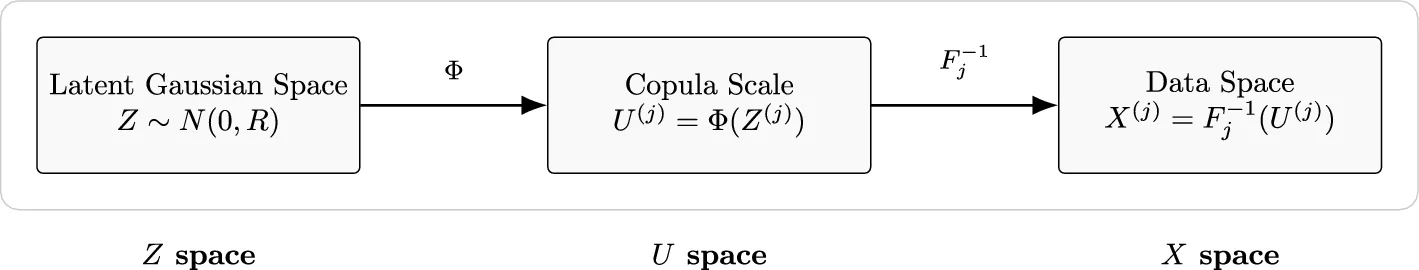
Three-stage transformation used by the copula anomaly generation procedure. A latent Gaussian vector *Z* determines dependence, the transformation U=Φ(Z) places each coordinate on a common uniform scale, and the inverse marginal CDFs map back to the data space *X*. Perturbing different stages of this pipeline yields the warp, joint, and support anomaly modes

We model dependence with a Gaussian copula [63, 64]. A latent Gaussian vector $Z=(Z^{(1)},\ldots ,Z^{(d)})$ is generated as Z∼N(0,R), where *R* is estimated from the training data, and $U^{(j)}=\Phi (Z^{(j)})$, j=1,…,d. This gives the three-stage transformation (Figure 4)

$$ Z \longrightarrow U \longrightarrow X, $$

where *Z* controls dependence, *U* places coordinates on a common uniform scale, and the inverse marginals map back to the observed data space. Perturbing different stages of this pipeline yields the three anomaly modes summarized in Table 3.

The three anomaly modes are designed to test different ways a tabular distribution can depart from the training data. The warp mode changes marginal tail behavior by distorting the uniform variables before mapping back to the original coordinate scales. This produces observations that remain tied to the estimated dependence structure but have altered marginal behavior. The joint mode perturbs the latent Gaussian representation, creating points in atypical regions of the dependence structure while preserving the original marginal mapping. The support mode moves a latent training point outward and then maps it through modified inverse CDFs that extrapolate beyond the observed coordinate ranges.

**Table 3:** Copula anomaly modes used in the experiments

Mode	Generation and labeling
warp	Draw z∼N(0,R) and set $u^{(j)}=\Phi (z^{(j)})$. Apply the coordinatewise warp $u^{*(j)} = \{u^{(j)}\}^{\gamma }/ [\{u^{(j)}\}^{\gamma }+\{1-u^{(j)}\}^{\gamma }]$ with γ>1, and map back by $x^{*(j)}=F_j^{-1}(u^{*(j)})$. The label is $a^*=1\{d_M(x^*)>\tau _{1-q}\}$, where $z^*=\Phi ^{-1}(u^*)$ and $d_M(x^*)=\{z^{*\top }R^{-1}z^*\}^{1/2}$.
joint	Choose a latent training point $z_b$, draw a random unit vector ζ, and sample $r=\sqrt{Q}$ with $Q\sim \chi ^2_d$ truncated to $[\chi ^2_d(1-q),\infty )$. Set $z^*=z_b+r\zeta$ and map back by $x^{*(j)}=F_j^{-1}\{\Phi (z^{*(j)})\}$. These points perturb the dependence structure while preserving the marginal mapping, and are labeled anomalous, $a^*=1$.
support	Choose a latent training point $z_b$ with $r_b=\{z_b^T R^{-1}z_b\}^{1/2}$. Draw $r^*=\sqrt{Q^*}$ from the upper (1-q) tail of $\chi ^2_d$ and set $z^*=z_b(r^*/r_b)$. Map back using a modified inverse CDF, $x^{*(j)}=\tilde{F}_j^{-1}\{\Phi (z^{*(j)})\}$, which extrapolates beyond the empirical support. These points are labeled anomalous, $a^*=1$.

Here $z_i=\Phi ^{-1}\{F(x_i)\}$ denotes the latent Gaussian representation of training point $x_i$, *q* is the tail probability, and $\tau _{1-q}$ is the empirical (1-q) percentile of $d_M(x_i)=\{z_i^T R^{-1} z_i\}^{1/2}$ over the training data (all experiments use q=0.05). The value $a^*$ is a binary label with value 1 for anomalous data

### Benchmarking on diverse real and simulated datasets

For benchmarking we used a curated subset of regression datasets from the Penn Machine Learning Benchmark (PMLB) repository [65, 66]. Only datasets with at least 10 features and a continuous response were retained, resulting in 61 datasets with dimensions ranging from d=10 to 124 and sample sizes from n=47 to 1066.

#### Experimental settings

Each dataset was randomly split into 80% training and 20% testing. Synthetic anomalies were generated using the copula-based procedure described in Section 5.2, with the number of anomalies matched to the number of test points. Each copula mode (warp, joint, support) was applied independently. To ensure that perturbations produced meaningful distributional shifts, the copula transformations were applied only to features identified as predictive of the outcome by selecting the top 10% using gradient boosting, as in the Friedman simulation, while the remaining features were left unchanged. Performance was measured by AUC-PR, with all evaluations repeated over 100 independent runs.

#### Results

**Fig. 5:**
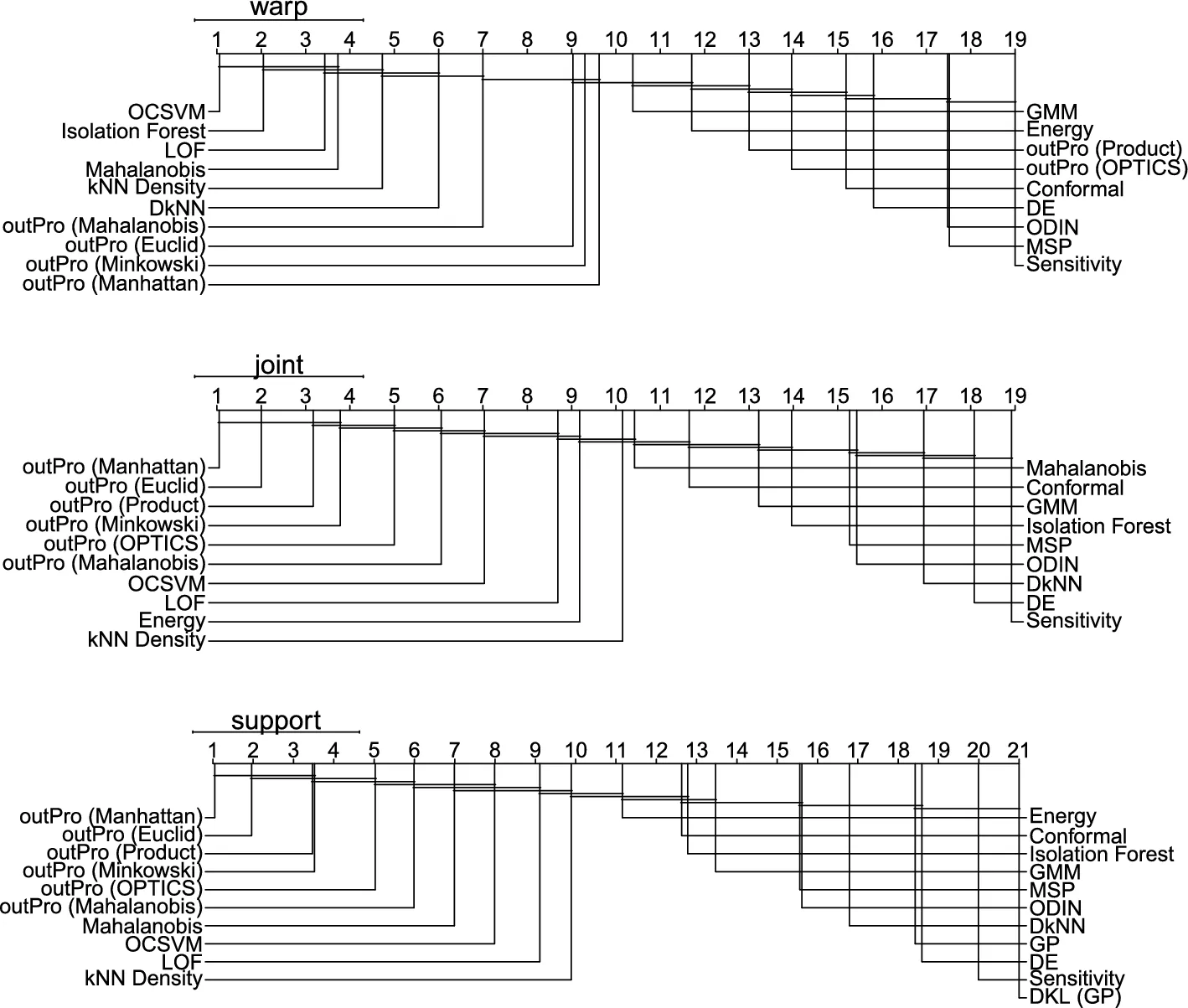
Critical difference (CD) plots comparing OOD detection methods across 61 PMLB regression datasets. From top to bottom, the panels correspond to the three anomaly generation modes, warp, joint, and support. Methods are ranked by average AUC-PR, and statistically indistinguishable groups at α=0.05 are connected by horizontal bars based on the Nemenyi test

Given the large number of datasets, we summarize performance by the average AUC-PR rank across datasets, shown as critical difference (CD) plots in Figure 5. Under the warp mode, the best methods are OCSVM, Isolation Forest, LOF, the global Mahalanobis score, robust *k*NN density, and DkNN. Next are the OutPro procedures, with Mahalanobis performing the best, followed by Euclidean, Minkowski, and Manhattan, while the product and OPTICS versions are lower. This pattern is reasonable because warp creates marginal tail distortion within the observed support and therefore favors methods driven by density, isolation, or global geometry.

Under the joint mode, the pattern changes significantly. The OutPro procedures are the best, with Manhattan first, followed by Euclidean, product, Minkowski, OPTICS, and Mahalanobis. The best non-OutPro methods are OCSVM and LOF, with energy, robust *k*NN density, and the global Mahalanobis score forming the next group. This shows that when anomalies alter dependence, methods built from local predictive profiles perform better than generic full-space scoring rules.

The same pattern holds for the support mode. OutPro procedures are again the best. Among the non-OutPro methods, global Mahalanobis score is the best, followed by OCSVM, LOF, robust *k*NN density, and energy, while conformal and Isolation Forest are in the next group. This shows that when anomalies exist outside the observed support, methods derived from local predictive profiles are especially effective.

### High-dimensional benchmarking

We next evaluate performance on five high-dimensional microarray datasets commonly used in the survival analysis literature, diffuse large B-cell lymphoma (DLBCL) [67], breast cancer [68], lung cancer [69], acute myeloid leukemia (AML) [70], and mantle cell lymphoma (MCL) [71].

**Table 4:** Summary of high-dimensional microarray datasets used for benchmarking after Cox-score filtering.

Dataset	*n*	*d*
AML	116	629
Breast Cancer	78	475
DLBCL	240	740
Lung Cancer	86	713
MCL	92	881

To place these in a regression framework, we converted survival outcomes into continuous pseudo-responses using random survival forests (RSF) [72]. RSF models were fit to each dataset, and out-of-bag (OOB) mortality predictions were extracted for use as regression targets. Although OutPro can handle survival data directly, this transformation enabled direct comparison with the baseline methods. To improve the signal-to-noise ratio, features were filtered by Cox-score ranking [73]. Table 4 summarizes sample sizes and dimensions for the filtered datasets.

#### Experimental settings

The experimental design mirrored that of the PMLB analysis. Each dataset was split into 80% training and 20% test sets. Synthetic anomalies were generated using the copula-based procedure under all three modes, with the number of anomalies matched to the number of test points. Anomaly perturbations were restricted to the top 10% of features identified as predictive by gradient boosting, while the remaining features were left unchanged. Each configuration was repeated over 100 independent runs, and performance was measured using AUC-PR. Due to the high-dimensional setting, the global Mahalanobis baseline could not be evaluated directly because the sample covariance matrices were singular, and therefore is omitted.

#### Results

**Fig. 6:**
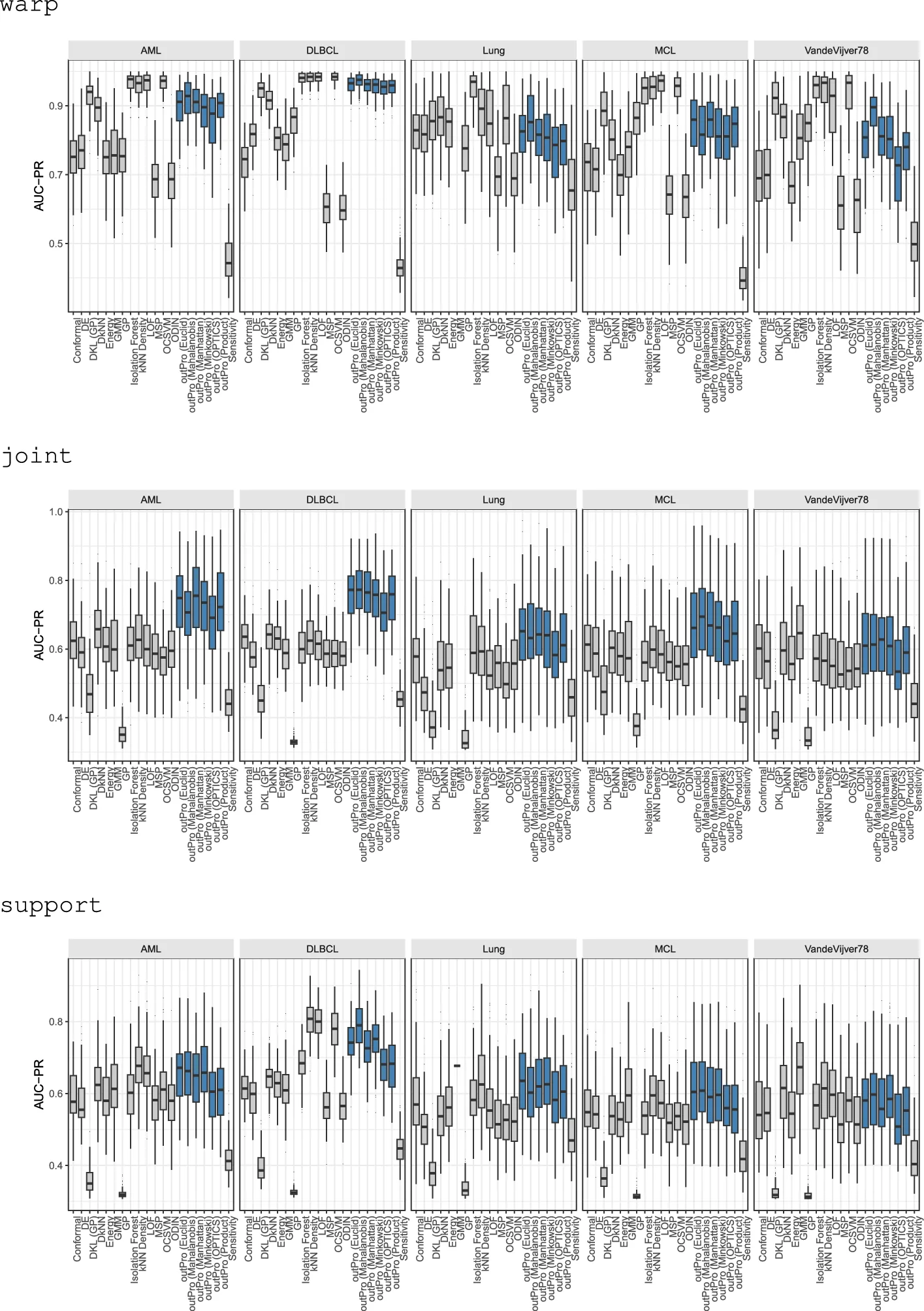
AUC-PR scores for high-dimensional microarray datasets under the three copula-based anomaly modes, warp, joint, and support. Each box summarizes results over 100 replications. Blue signifies OutPro methods

**Fig. 7:**
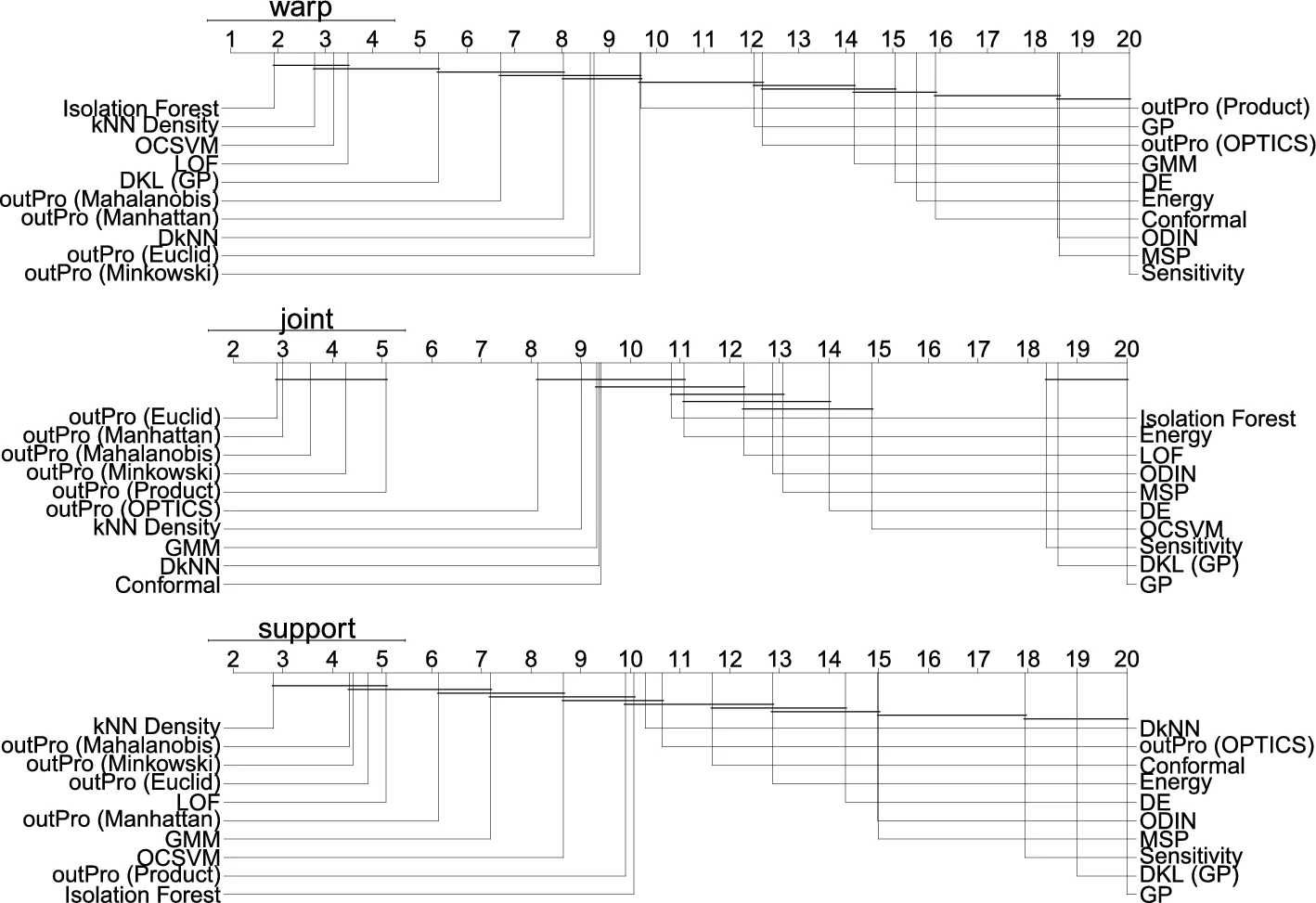
Critical difference (CD) plots showing average rank performance across the five high-dimensional microarray datasets and copula modes. Lower ranks indicate better performance. The warp panel is led by Isolation Forest, robust *k*NN density, OCSVM, and LOF. The joint panel is led by OutPro distances. The support panel is more mixed, with robust *k*NN density and several OutPro distances near the top

Boxplots of AUC-PR by dataset and copula mode are shown in Figure 6, with an overall summary via critical difference (CD) plots given in Figure 7. The ranking pattern is more mixed than in the PMLB analysis.

**Fig. 8:**
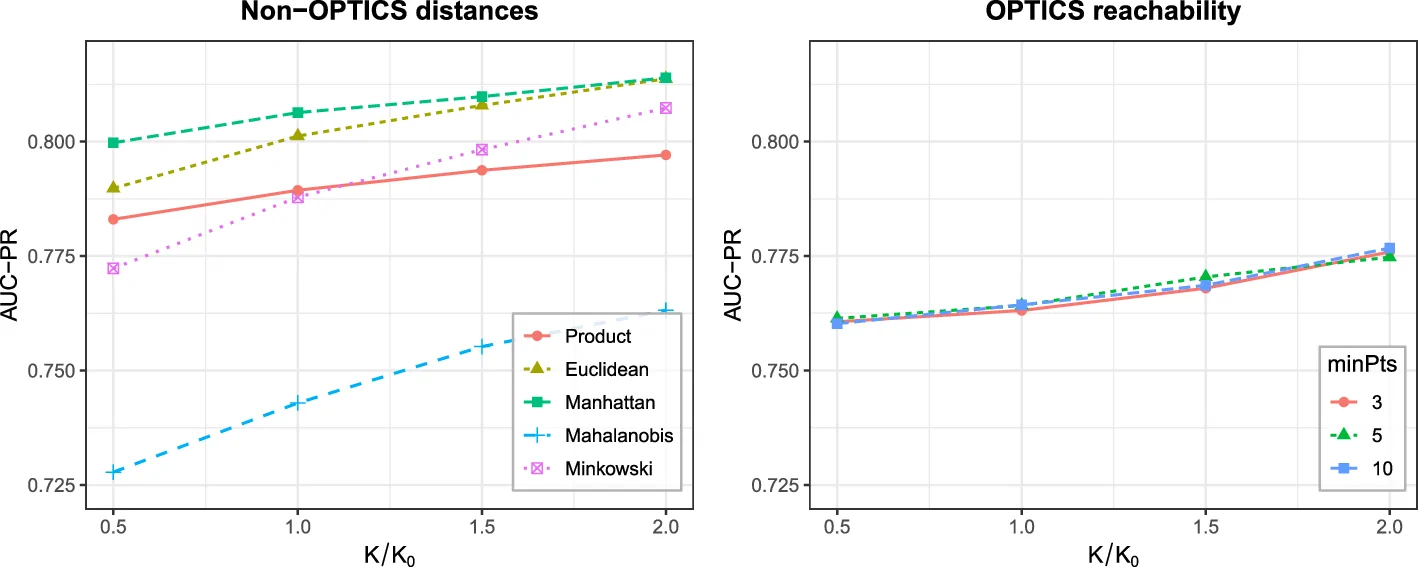
Sensitivity of OutPro to neighborhood size *K* under the support anomaly mode on the 61 PMLB regression datasets. The left panel shows AUC-PR for the non-OPTICS distances as a function of $K/K_0$. The right panel shows AUC-PR for the OPTICS reachability score for different values of $\texttt {minPts}$

Under the warp mode, the best methods are Isolation Forest, robust *k*NN density, and OCSVM, while LOF and DKL (GP) also perform well. Among the OutPro procedures, Mahalanobis is best, while Manhattan, Euclidean, Minkowski, and product fall somewhere in the middle of all procedures. This again fits the structure of warp, which generates tail distorted points that remain largely within the observed support and can therefore be captured well by classical density based rules.

As in the previous PMLB study, the picture changes under the joint mode. OutPro Euclidean, Manhattan, Mahalanobis, Minkowski, product, and OPTICS are the top procedures. The best non OutPro methods are robust *k*NN density, GMM, DkNN, and conformal prediction. Thus when anomalies act through dependence rather than marginal extremes, the local predictive structure used by OutPro is more informative than generic density or uncertainty scores.

Under the support mode, the results are mixed. Robust *k*NN density has the best performance. LOF is also near the top. At the same time, OutPro Mahalanobis, Minkowski, Euclidean, and Manhattan occupy four of the six top spots, showing that OutPro remains highly competitive. The product based OutPro score is less effective, especially under support, where it ranks below the other OutPro methods. One explanation is that in these high-dimensional datasets many coordinates carry signal, so a multiplicative combination of coordinate wise discrepancies can amplify noise and dilute informative deviations. Additive or correlation adjusted distances such as Manhattan, Euclidean, Minkowski, and Mahalanobis appear more stable in this setting.

### Sensitivity analysis for *K* and minPts

To assess the stability of OutPro with respect to its main tuning parameters, we carried out a focused ablation on the PMLB regression datasets used in Section 5.3. We chose the support copula mode for this analysis because it was the most challenging setting in the benchmark. Recall from Section 3 that *K* is the number of training points used for the neighborhood $\mathcal {N}_K(x)$. By design, OutPro uses values of *K* that are much larger than those typical of classical nearest-neighbor methods. The default neighborhood size used in our previous analyses was $K_0 = \min (n/10, 5000)$; here we evaluated *K* such that $K/K_0 \in \{0.5, 1, 1.5, 2\}$. For the OPTICS distance we also varied $\texttt {minPts} \in \{3, 5, 10\}$. Because $\texttt {minPts}$ enters only the OPTICS reachability calculation, the non-OPTICS distances were summarized after averaging over $\texttt {minPts}$, while OPTICS was reported separately for each value.

#### Results

Figure 8 summarizes the AUC-PR values. Overall, we observe that every non-OPTICS distance attained its best AUC-PR at the largest neighborhood (here equal to $2K_0$), with the Manhattan, Euclidean and Minkowski distances achieving best overall performance. When using OPTICS, AUC-PR similarly improved with *K* but was essentially flat across $\texttt {minPts} \in \{3, 5, 10\}$.

This shows OutPro is stable across its tuning parameters. The default value $K_0$ worked well, and while larger neighborhoods improved performance, the gains were moderate. The monotone improvement with *K* is consistent with the design of the method. Because $\mathcal {N}_K(x)$ is constructed from the fitted model’s rule regions rather than from a global distance, it already concentrates on training cases that are functionally relevant to *x*. Enlarging *K* within this model-derived neighborhood adds useful context without introducing the noise that would arise from expanding a conventional nearest-neighbor search.

### Component ablation

We next isolate the main components of OutPro using the PMLB regression benchmark. The components are the VarPro signal set $\widehat{S}$, the forest neighborhood $\mathcal {N}_K(x^*)$, and the VarPro weights used in the final distance. Here the forest neighborhood means the set of *K* training points with the largest OutPro proximity scores $W(x_i)$ for the test input $x^*$, as defined in Sect. 3.3. Ordinary KNN is the set of *K* training points with the smallest Manhattan distance to $x^*$, computed using the coordinates and weights specified in Table 5.

To keep the ablation focused on algorithmic components, all settings use the Manhattan distance and the default neighborhood size $K_0=\min (n/10,5000)$. Table 5 reports average AUC-PR ranks across the PMLB regression benchmark. Lower ranks indicate better performance.

**Table 5:** Component ablation on the PMLB regression benchmark. Lower average AUC-PR rank is better. The column $\bar{r}$ is the average rank across the three copula modes.

Setting	Space	Neighborhood	Weights	joint	support	warp	$\bar{r}$
Full OutPro	$\widehat{S}$	forest	VarPro	2.88	1.40	1.23	1.84
Unweighted OutPro	$\widehat{S}$	forest	uniform	3.38	3.42	3.80	3.53
All-coordinate OutPro	all	forest	VarPro	2.91	2.94	3.89	3.25
Subspace KNN	$\widehat{S}$	ordinary KNN	VarPro	1.47	2.82	2.30	2.33
Full-space KNN	all	ordinary KNN	uniform	4.37	4.42	3.78	4.19

The results support the integrated construction of OutPro. Full OutPro has the best average rank across the three copula modes, with especially strong performance for support and warp shifts. Removing VarPro weights while keeping the same selected variables and forest neighborhood reduces performance in all three modes. Removing the subspace restriction by using all coordinates also reduces performance, consistent with signal dilution from nuisance coordinates.

The comparison with KNN shows that the effect of the forest neighborhood depends on the type of shift. Subspace KNN has the best rank for joint shifts, but Full OutPro is substantially better for support and warp shifts and is better overall. Full-space KNN performs poorly in all settings, indicating that ordinary nearest-neighbor search in the full covariate space is not sufficient.

## Evaluating lymphadenectomy survival through OOD analysis

We next consider a survival case study from the Worldwide Esophageal Cancer Collaboration (WECC) [74]. The registry includes patients who underwent esophagectomy alone for esophageal cancer, with right censored follow-up on all-cause mortality [75]. Previous WECC analyses used random survival forests (RSF) [72] to study the relation between lymphadenectomy, measured by the number of lymph nodes resected, and five-year survival. Those analyses found that greater lymphadenectomy was generally associated with improved survival, with optimal counts depending on tumor stage and histopathologic type [75]. We revisit this setting to study whether patients receiving more extensive lymphadenectomy become less outlying relative to clinically relevant training populations.

The analysis used $n=6{,}142$ adenocarcinoma cases. Covariates included pTNM classification, number of lymph nodes resected, number of positive nodes, histologic grade, tumor location and length, residual cancer status, and patient demographics, giving d=35 variables. In the pTNM system, the prefix “p” denotes pathologic staging from surgical resection specimens, and the T category records depth of tumor invasion from pT1 to pT4. We focus on patients with pT3 or pT4 tumors, combined into a single group.

**Fig. 9:**
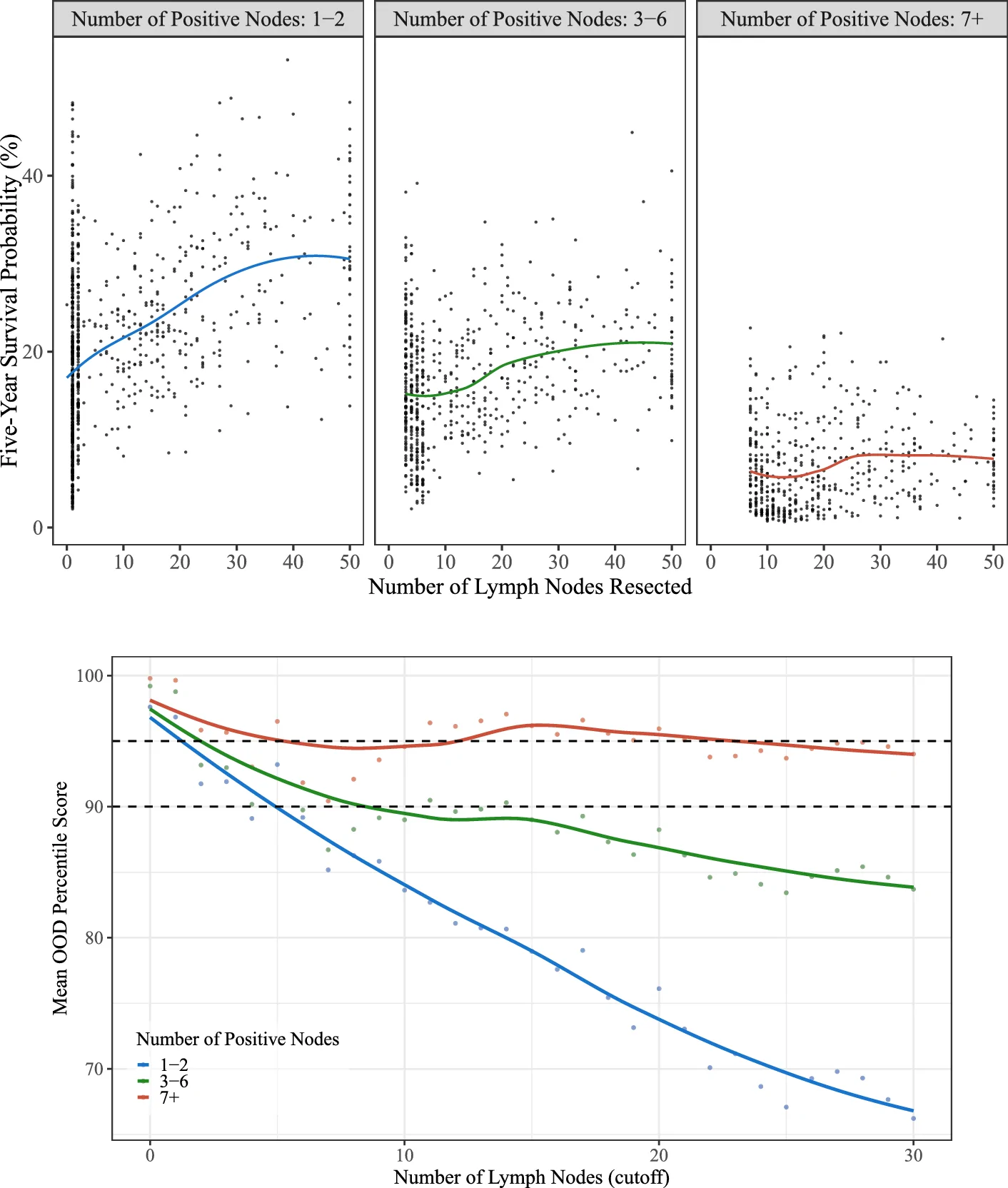
WECC adenocarcinoma analysis for pT3–4 tumors. Upper panel shows out-of-bag five-year survival predictions from RSF as a function of the number of lymph nodes removed, stratified by the number of positive nodes. Survival generally improves with greater lymphadenectomy, with smaller gains as nodal involvement increases. Lower panel shows mean OOD percentile score as a function of the lymphadenectomy cutoff, for each of 31 hold-out scenarios with cutoffs from 0 to 30. A score of 100 corresponds to the most extreme OOD score under the null training distribution

The upper panel of Fig. 9 gives the RSF out-of-bag five-year survival predictions for pT3–4 adenocarcinoma, stratified by the number of positive nodes, 1–2, 3–6, and ≥7. The pattern recovers the clinical signal reported previously: survival improves as more lymph nodes are removed, but the gain is attenuated in patients with heavier nodal involvement.

For OOD scoring, we created 31 train/test scenarios by varying the lymphadenectomy cutoff from 0 to 30 nodes. At each cutoff, the test set contained node-positive pT3–4 patients whose lymph node count met or exceeded the cutoff, and all remaining patients formed the training set. A cutoff of 0 therefore holds out all node-positive pT3–4 cases, a clinically distinct group with deeply invasive disease and nodal involvement. As the cutoff increases, the held-out group is restricted to patients with progressively greater lymphadenectomy. For each scenario, we computed the mean OOD percentile score for the held-out group on a 0–100 scale.

The lower panel of Fig. 9 shows that mean OOD scores decrease as the cutoff increases, indicating that patients with greater lymphadenectomy become less distinct from the corresponding training population. The decline is steepest for patients with 1–2 positive nodes and shallowest for those with ≥7 positive nodes. This is consistent with the survival curves in the upper panel, where the benefit of additional lymphadenectomy is less pronounced with heavier nodal involvement. Using the 95th percentile as a threshold for considering a case non-OOD, the required lymphadenectomy count is approximately 3 nodes for the 1–2 and 3–6 positive-node categories, but about 30 nodes for the ≥7 category. At a stricter 90th percentile threshold, the required count rises to 5 and 10 nodes for the 1–2 and 3–6 categories, respectively.

Because the surgeon does not know the true nodal status during resection, the OOD analysis supports a more aggressive lymphadenectomy for suspected deeply invasive tumors, on the order of 30 nodes. This finding is consistent with the earlier WECC report, which estimated optimal lymphadenectomy counts for pT3/T4 cancers ranging from 29 to 50 nodes depending on histopathologic type [75].

## Summary and limitations

We have proposed an integrated model-aware, subspace-aware framework for OOD detection in supervised settings. A key feature of the method is that the OOD score is not constructed from the model’s predicted values, residuals, or uncertainty measures, but rather from the rule structure learned by a supervised forest. Those learned rules identify a reference neighborhood for each test input and select a predictive subspace, after which OOD is measured by distances computed only within that subspace. The fitted model therefore enters the detector through its learned partition of the covariate space rather than through its output, which focuses the score on covariate variation that is relevant for the outcome and separates functionally meaningful departures from shifts occurring mainly in irrelevant coordinates. The framework is general, and the empirical work in this paper focused on regression and survival settings.

The empirical results show that performance depends on the form of the shift. In the Friedman experiments, OutPro performed best for small feature-targeted perturbations, where anomalies are subtle and difficult to distinguish from ordinary test variation. In the PMLB benchmarks, OutPro was best under the joint and support modes. Under the warp mode, however, classical tabular methods such as Isolation Forest, one-class SVM, Local Outlier Factor, and robust *k*NN density were often among the best procedures. In the high-dimensional microarray studies, OutPro again performed very well under joint and remained competitive under support, while the warp results were more mixed. This pattern is consistent with the structure of warp, which creates low-density marginal tail distortion within the observed support and is therefore well matched to density and isolation rules. A sensitivity analysis also suggested that the method is reasonably stable over a broad range of its key tuning parameters, including *K* (the number of neighbors), with only moderate gains from enlarging the model-derived neighborhood.

The case study on esophageal cancer and lymphadenectomy illustrated the clinical interpretability of the resulting scores. Using pT3-4 adenocarcinoma patients from the WECC dataset, we examined how increasingly aggressive lymphadenectomy moved patients toward or away from anomalous regions relative to the training cohort. The analysis suggested that for suspected deeply invasive tumors, removal of approximately 30 lymph nodes may be a reasonable surgical target when true nodal status is unknown, in line with prior WECC findings.

OutPro was also computationally manageable across all studies considered. Runtimes across the PMLB regression benchmarks and the high-dimensional microarray datasets were well under 30 seconds at their largest, and in the microarray studies were especially short because variable prioritization substantially reduced the working dimension and sample sizes were moderate. The computation benefits from three features of the procedure, namely that the prediction engine is based on random forests, that priority rules can be extracted efficiently from the trained ensemble, and that the final neighborhood scoring step is inexpensive once the predictive subspace has been selected.

Several limitations should be mentioned. First, the empirical results show that no single detector is uniformly best, and within OutPro no single subspace distance dominates across all settings. The product score worked well in lower-dimensional settings, while Manhattan, Euclidean, Minkowski, and Mahalanobis scores were often more stable in higher-dimensional problems, and classical tabular methods were especially competitive under warp. A fuller theory for choosing the subspace distance and identifying the settings where each score is preferred remains open. Second, the method depends on estimating a predictive subspace from the fitted model, and errors in that step can affect both the reference neighborhood and the final score. Third, while the copula-based benchmarks provide varied and controlled departures, any synthetic benchmark remains an approximation to the shifts encountered in practice. A fourth limitation concerns pure concept shift. Although the trained forest, signal variables, and reference neighborhoods are learned from labeled training data, the OutPro score for a new input is computed from its covariates. Consequently, two test distributions with the same $\mathbb {P}^*_X$ but different $\mathbb {P}^*_{Y\mid X}$ induce the same distribution of OutPro scores. The method is therefore intended for prediction relevant covariate departures, not for detecting conditional shifts that leave the covariate distribution unchanged.

## Data Availability

Our code is publicly available as a CRAN R-package varPro. All simulated datasets can be reproduced directly from the code. Public benchmark datasets used in this study are available from the PMLB repository, and information on the microarray datasets can be found in the cited references. The esophageal cancer data from the Worldwide Esophageal Cancer Collaboration are not publicly available.
